# Counter-Intuitive Features of Particle Dynamics in Nanopores

**DOI:** 10.3390/ijms242115923

**Published:** 2023-11-03

**Authors:** Alexander M. Berezhkovskii, Sergey M. Bezrukov

**Affiliations:** Section on Molecular Transport, *Eunice Kennedy Shriver* National Institute of Child Health and Human Development, National Institutes of Health, Bethesda, MD 20892, USA; alexanderb@mail.nih.gov

**Keywords:** nanopore sensing, membrane transport, metabolite channels, diffusion, Smoluchowski equation, channel gating, selectivity

## Abstract

Using the framework of a continuous diffusion model based on the Smoluchowski equation, we analyze particle dynamics in the confinement of a transmembrane nanopore. We briefly review existing analytical results to highlight consequences of interactions between the channel nanopore and the translocating particles. These interactions are described within a minimalistic approach by lumping together multiple physical forces acting on the particle in the pore into a one-dimensional potential of mean force. Such radical simplification allows us to obtain transparent analytical results, often in a simple algebraic form. While most of our findings are quite intuitive, some of them may seem unexpected and even surprising at first glance. The focus is on five examples: (i) attractive interactions between the particles and the nanopore create a potential well and thus cause the particles to spend more time in the pore but, nevertheless, increase their net flux; (ii) if the potential well-describing particle-pore interaction occupies only a part of the pore length, the mean translocation time is a non-monotonic function of the well length, first increasing and then decreasing with the length; (iii) when a rectangular potential well occupies the entire nanopore, the mean particle residence time in the pore is independent of the particle diffusivity inside the pore and depends only on its diffusivity in the bulk; (iv) although in the presence of a potential bias applied to the nanopore the “downhill” particle flux is higher than the “uphill” one, the mean translocation times and their distributions are identical, i.e., independent of the translocation direction; and (v) fast spontaneous gating affects nanopore selectivity when its characteristic time is comparable to that of the particle transport through the pore.

## 1. Introduction

Membrane channels that allow metabolites and other macromolecular solutes to exchange between cells or different cell compartments are protein structures forming water-filled nanopores. Traditionally, large channels were thought to be “molecular sieves”, which discriminate between different solutes based entirely on their size. In other words, they were regarded as low-selectivity filters that allow for the passage of small enough solutes without any specific interaction with the channel nanopore. However, existing and accumulating experimental evidence [1,2,3,4,5,6,7,8,9] indicates that many large channels exhibit such interactions with the solutes they transport. It appears that the channel nanopore linings are tuned by evolution towards the preferential transport of certain solutes. Therefore, to understand large channel functioning, the size-exclusion principle should be complemented by considering the particle-channel interactions.

Basic transport properties of a channel, most importantly, its solute selectivity and transport rates, are indeed controlled by the interactions of solute molecules with the channel walls. In a theoretical analysis, such interactions are often modelled either by employing a series of discrete binding sites [10,11,12,13,14,15,16,17,18] or by a continuous diffusion model based on the one-dimensional Smoluchowski equation with solute-channel interactions represented by a potential of mean force [19,20,21,22,23,24]. This potential not only accounts for the Coulomb, van der Waals, and other “direct” forces between the solute molecule and nanopore wall but also may include an entropy term that takes care of the changing confinement geometry due to a coordinate-dependent pore radius. The two models, the discrete and continuous diffusion ones, can be mapped onto each other [11,23].

**Figure 1:**
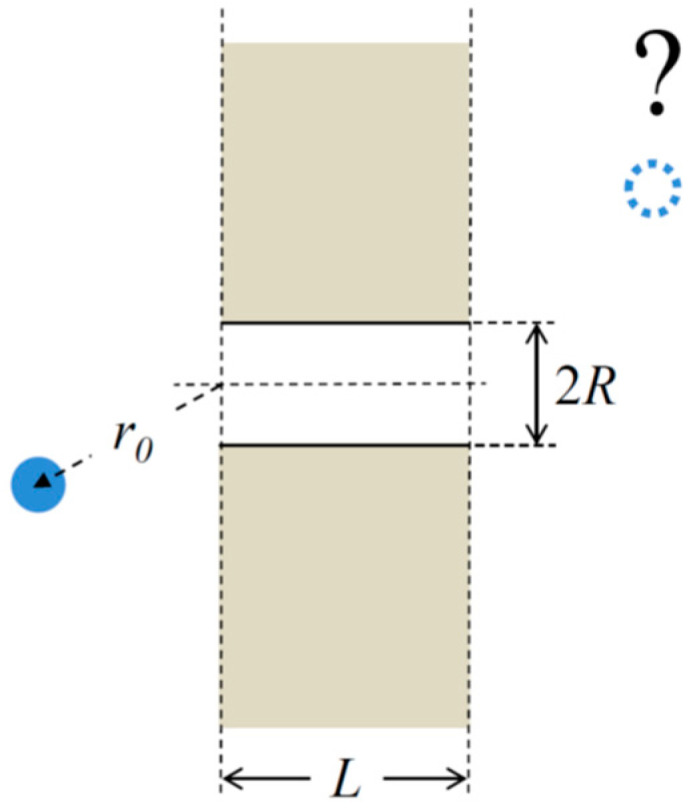
In the simplest, idealized case, a single transmembrane nanopore is represented by the right cylinder of a constant radius R and length L. What is the probability for a particle, starting at distance $r_{0}$ from the pore opening, to pass through the pore and escape to infinity on the opposite side of otherwise impermeable membrane?

For a physicist studying transport through membrane channels, the most immediate set of questions to start with are those of probability and characteristic times of channel-facilitated particle translocation. It turns out that even for the highly simplified case schematically illustrated by Figure 1, which does not involve any specific interactions between the particle and channel, the role of nanopore geometric parameters and particle bulk and intra-channel diffusivities could be rather non-trivial.

One may naturally expect that a wider pore would offer a higher translocation probability, while a pore of an increased length would make it smaller. This is true, but is there an analytical description that relates this probability to the channel geometry? As shown below, for the translocation problem illustrated in Figure 1 with equal particle diffusivities in the bulk and in the channel, the translocation probability is given by the following:

(1) $$P_{tr}^{bulk}≅\frac{R}{r_{0}\left(\pi +\frac{2L}{R}\right)},\;r_{0}\geq 2R,$$

where $P_{tr}^{bulk}$ is the probability of a particle, starting in the bulk at distance $r_{0}$ from the center of the left opening of the pore, to translocate through a straight cylindrical pore of radius R and length L and to escape to infinity in the right reservoir. Equation (1) shows that the probability is indeed increasing with the pore radius and decreasing with the pore length. Such dependence is, most probably, in comfortable accord with one’s intuition.

Less intuitive questions are (a) what happens when the particle diffusivity in the channel differs from that in the bulk and (b) how particle specific interactions with the pore wall affect the transport. In what follows, we will give answers to this kind of questions, including a number of counter-intuitive ones, which are the main topic of the present article. Five examples to be considered here in detail are as follows:

  (i). Attractive interactions with the pore walls create a potential well inside the pore, which makes a particle spend more time in the channel but increases the particle flux.
 (ii). If the potential well-describing particle-channel interaction occupies only part of the pore length, the mean translocation time is a non-monotonic function of the well width, first increasing and then decreasing with it.
(iii). For a rectangular potential well occupying the entire pore, the mean residence time of a particle in the pore is independent of its intra-channel diffusivity and depends only on its diffusivity in the bulk.
 (iv). In the presence of a potential bias, the “downhill” particle flux is, as might be expected, higher than the “uphill” one; however, the mean particle downhill and uphill translocation times (as well as their distributions) are identical.
  (v). Stochastic gating, if it is fast enough, provides a new selectivity mechanism that enhances relative transport rates of slower moving particles by orders of magnitude.

Although our analysis below is based on the continuous diffusion model of solute dynamics in the channel, similar consideration can be performed in the framework of the stochastic site model of the intra-channel dynamics. The reason is that such a model may be viewed as a course-grained version of its continuous diffusion counterpart.

From the very beginning, we must point out that our approach differs from most of those in the current literature. We use a lot of drastic simplifications concerning the geometry of the nanopore—usually accepting that of a right circular cylinder—and the multiple particle-nanopore interactions of a different origin that are modeled by a one-dimensional potential of mean force, usually of a rectangular shape. We are aware that the list of the multiple interactions, in particular, includes electrostatic screening and energy barriers experienced by charged particles entering channels in membranes with a low-dielectric constants [25,26,27], van der Waals, Coulomb, and hydrophobic interactions between solutes and the pore walls [2,28,29,30] or even more elaborated interactions, such as the reversible binding of substrates to enzymes [31]. An additional complication is that the potential of mean force has to account for different hydrodynamic effects [32,33], including those of electroosmosis [34,35,36]. The channel-facilitated transport can also be substantially modified by the channel dynamics [37] and the channel pore response to the permeating ion [38].

Though all these interactions “thrown in one basket” can rarely be represented by any simply shaped potential of mean force, the radical simplifications allow us to obtain a quantitative, sometimes even algebraic, description of many features of the particle intra-channel dynamics. It demonstrates how the confinement and specific interactions with nanopores affect these features. In what follows, we will discuss the five examples listed above in detail sufficient to understand them both quantitatively and, most importantly, qualitatively. We also give all the necessary references to the articles presenting original derivations.

## 2. Results

We analyze channel-facilitated transport in the framework of the one-dimensional Smoluchowski equation augmented by radiation boundary conditions at the channel pore openings to account for the fact that the problem is three-dimensional [19]. To make the present article self-contained, the approach is briefly summarized in Appendix A, where the choice of the boundary conditions is rationalized. Specifically, it is explained that they are determined by the requirement of detailed balance at the two channel openings.

One of the most important questions is indeed the question of the precision and accuracy of such a framework for the description of the actual three-dimensional problems. The question of the accuracy of our approach was addressed in a number of Brownian dynamic simulations, in which Brownian particles performed three-dimensional motion not only in the bulk, but also inside the nanopore. A good agreement between theoretical predictions and simulation results was demonstrated, with deviations not exceeding several percent. Specifically, the predictions for the particle translocation probability vs. simulation experiments were compared in Refs. [20,39] and those for the particle residence and capture times in Refs. [21,39].

Here, we mostly recapitulate analytical results obtained previously to highlight our major findings. We represent molecule interaction with the channel pore by the potential of mean force, which allows us to study the kinetics of molecular exchange between the bulk and the pore, to evaluate the consequences of inter-molecular interactions and to analyze the effects of fast gating even when time characteristics of the gate dynamics are comparable to those of solute diffusion in the channel.

**Figure 2:**
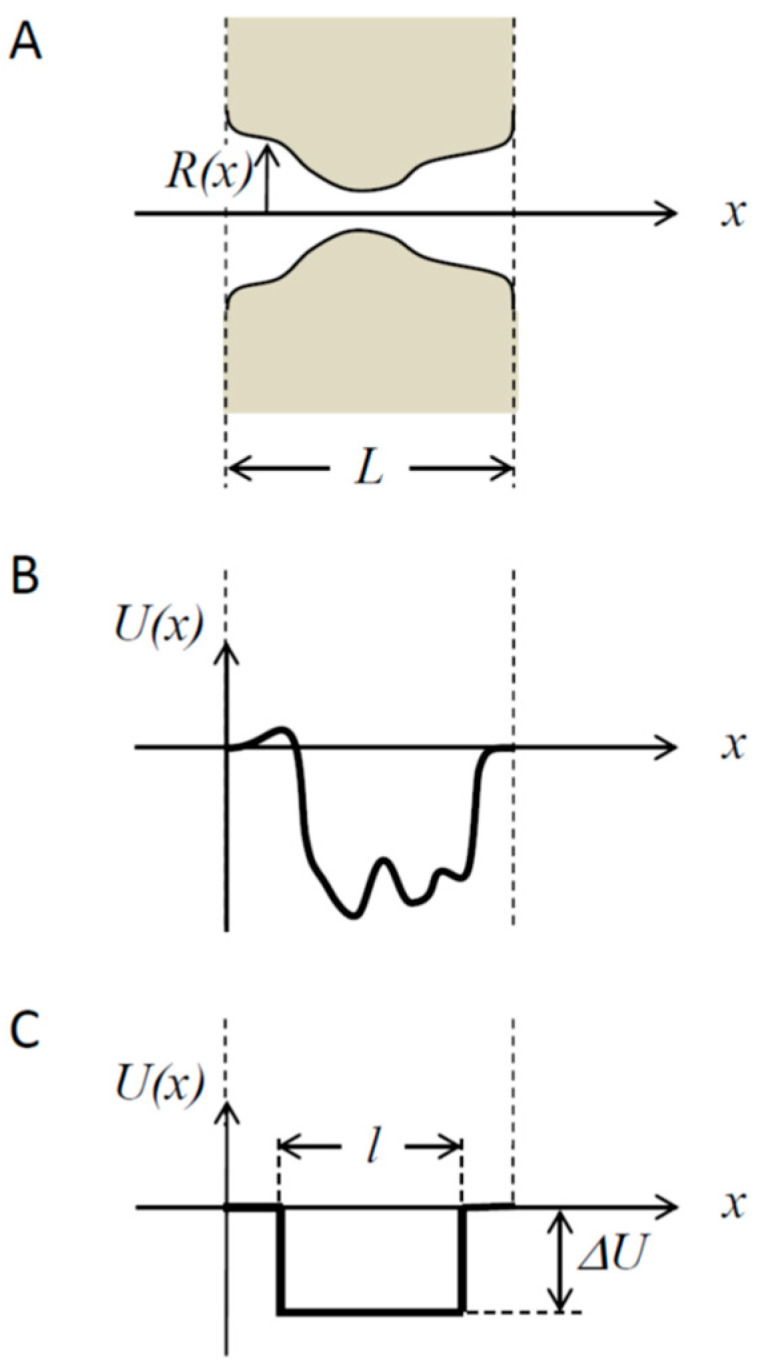
A more realistic representation of the channel structure (panel (**A**)) includes a coordinate-dependent radius $R\left(x\right)$. The particle-channel interactions are characterized by a coordinate-dependent potential of mean force $U\left(x\right)$, which, in addition to the obvious entropy contributions, may also include Coulomb, van der Waals, and other types of specific interactions between the translocating particle and pore walls (panel (**B**)). A simplified version of the potential of mean force (panel (**C**)) that allows us to obtain simple analytical expressions for quantities characterizing particle transport through the pore containing a rectangular potential well of depth ΔU occupying a fraction l/L of the total channel length L.

A still simplified but more realistic structure of a transmembrane channel is illustrated in Figure 2A. It shows a channel pore of a varying cross-section characterized by a coordinate-dependent radius $R\left(x\right)$, which leads to the entropic contribution [40,41] to the potential of mean force $U\left(x\right)$ given by $-k_{B}T\ln[R\left(x\right)/R(0)]$, with $k_{B}$ and T having their usual meaning of the Boltzmann constant and absolute temperature, respectively. An example of an arbitrary potential $U\left(x\right)$ is given in Figure 2B. A special shape of a rectangular potential well of depth ΔU occupying only part of the pore of length l, l<L, is shown in Figure 2C. The choice of such a well may look somewhat artificial, but it roughly mimics the particle–pore interaction and allows us to derive analytical results in a relatively simple form, thus facilitating their analysis and leading to important qualitative conclusions of the general consequence formulated below.

### 2.1. Translocation Probability and Steady-State Flux

Recently, we have studied, both analytically and in Brownian dynamic simulations, trapping the kinetics of single particles diffusing in a half-space bounded by a reflecting flat surface containing an absorbing circular disk [39]. We have shown that within the accuracy of several percent, the probability $P_{trap}^{dics}$ of particle trapping by the disk of radius R can be described by an absorbing hemisphere approximation wherein the probability depends only on the distance $r_{0}$ between the particle starting point and the disc center and not on the starting point angle. This is true on the condition that $r_{0}\geq 2R$. The result for the trapping probability is as follows:

(2) $$P_{trap}^{dics}≅\frac{2R}{\pi r_{0}}.$$

Earlier, we derived a general expression for the translocation probability $P_{tr}^{ch}$ of a particle that is placed at the pore opening to translocate to the other side of the membrane and escape to infinity [20]. For the case of a zero potential drop across the channel (Figure 2B,C), U(0)=U(L)=0 and R(0)=R(L)=R, i.e., for the case of diffusion-driven transport, this probability is given by the following:

(3) $$P_{tr}^{ch}=\frac{1}{2+\frac{4D_{b}}{\pi R}\int_{0}^{L}\exp\left[\beta U(x)\right]\frac{dx}{D_{ch}(x)}},$$

where $D_{ch}(x)$ is the coordinate-dependent intra-channel diffusivity and $\beta =1/(k_{B}T)$. Combining these results, we obtain the following expression for the particle “bulk-to-bulk” translocation probability:

(4) $$P_{tr}^{bulk}≅\frac{R}{\pi r_{0}+\frac{2D_{b}r_{0}}{R}\int_{0}^{L}\exp\left[\beta U(x)\right]\frac{dx}{D_{ch}(x)}},\;r_{0}\geq 2R.$$

In the case of a straight cylindrical pore shown in Figure 1 with U(x)=0 and $D_{ch}(x)=D_{b}$, we recover Equation (1). The expression in Equation (4) is more informative as it allows one to analyze the effects of the intra-channel potential of mean force and position-dependent diffusivity. It is easy to see that deep potential wells and very high diffusivities lead to the maximum value of the bulk-to-bulk translocation probability, which is given by $R/(\pi r_{0})$.

For the mean lifetime τ of a particle in the pore, before the particle escapes to infinity, conditional on the idea that (a) the particle starts from the left channel opening and (b) U(0)=U(L) and R(0)=R(L), we have derived the following [21]:

(5) $$\tau =\frac{\pi R}{4D_{b}}\frac{\int_{0}^{L}\left[1+\frac{4D_{b}}{\pi R}\int_{x}^{L}\frac{\exp\left[\beta U(y)\right]}{D_{ch}(y)}dy\right]\exp\left[-\beta U(x)\right]dx}{2+\frac{4D_{b}}{\pi R}\int_{0}^{L}\frac{\exp\left[\beta U(x)\right]}{D_{ch}(x)}dx}.$$

Importantly, the validity of expressions in Equations (2), (3) and (5) was verified by comparing them with the results of three-dimensional Brownian dynamic simulations [20,21,39].

We now consider, for simplicity, the case of a constant intra-channel diffusivity, $D_{ch}(x)=D_{ch}$, which may differ from its bulk counterpart and a rectangular potential well (Figure 2C) of depth ΔU occupying the entire pore, l=L.

(6) U(x)=−ΔUH(x)H(L−x),

where H(x) is the Heaviside step function. Expressions in Equations (3) and (5) then reduce to the following:

(7) $$P_{tr}=\frac{1}{2+\frac{4D_{b}L}{\pi D_{ch}R}\exp\left(-\beta \Delta U\right)}$$

and

(8) $$\tau =\frac{\pi RL}{8D_{b}}\exp\left(\beta \Delta U\right).$$

The accuracy of the above expressions was checked in three-dimensional Brownian dynamic simulations with the well depth ΔU varying in the range of 0 to 4.5 $k_{B}T$. It was established that the translocation probability, while changing by more than an order of magnitude with the varying depth, was in agreement with the analytics within 9% (Figure 3 of Ref. [20]); the mean lifetime in the pore was found to be within 3% of the analytical prediction while changing by two orders of magnitude (Figure 9 of Ref. [21]).

It is an interesting and quite counter-intuitive result that this time does not depend on the particle diffusivity in the channel pore. A detailed discussion of this finding and its relevance to single-molecule nanopore experiments is given in Ref. [42].

Using $P_{tr}$ in Equation (7), one can find the flux of non-interacting particles through the channel, $J_{ni}=k_{on}^{}\left(c_{L}-c_{R}\right)P_{tr}^{}$, where $k_{on}=4D_{b}R$ is the Hill–Berg–Purcell rate constant [43,44] (see Appendix A), and $c_{L}$ and $c_{R}$ are particle concentrations in the left and right bulk reservoirs, respectively.

Now, we account for the particle–particle interaction in the simplest case of a very strong inter-particle repulsion in the channel. This implies that the channel pore can be occupied by only one particle, so that the particle residing in the pore makes it inaccessible to other particles. Then, we use the following expression for the flux [45]:

(9) $$J=\frac{k_{on}^{}\left(c_{L}-c_{R}\right)P_{tr}^{}}{1+k_{on}\left(c_{L}+c_{R}\right)\tau },$$

with the denominator accounting for the channel single occupancy, and we obtain the following:

(10) $$J=\frac{2D_{b}R\left(c_{L}-c_{R}\right)}{\left[1+\frac{\pi R^{2}L\left(c_{L}+c_{R}\right)}{2}\exp\left[\beta U(x)\right]\right]\left[1+\frac{2D_{b}L}{\pi D_{ch}R}\exp\left[-\beta U(x)\right]\right]}.$$

An analysis of Equation (10) shows that the flux is a non-monotonic function of the well depth ΔU. The depth that maximizes the flux provides a compromise between sufficiently high translocation probability, which increases with the well depth and not too long blockage of the channel. The optimal depth is given by the following:

(11) $$\Delta U_{opt}=\frac{k_{B}T}{2}\ln\left[\frac{4D_{b}}{\pi^{2}D_{ch}R^{3}\left(c_{L}+c_{R}\right)}\right].$$

This depth is a function of the bulk concentrations of the translocating molecules. This is something to be expected because the flux magnitude is controlled by the interplay between two main parameters: the dwell time of the particle in the channel and the probability of particle translocation. As the well depth grows, the probability, according to Equation (7), increases. However, according to Equation (8), the dwell time increases too, and the presence of a particle in the channel blocks it for the entrance of the next one, thus decreasing the flux. Particle concentrations in the bulk determine the frequency of their attempts to enter the channel pore, $1/\tau_{\mathrm{emp}}=k_{\mathrm{on}}\left(c_{L}+c_{R}\right)=4D_{b}R\left(c_{L}+c_{R}\right)$. As $c_{L}+c_{R}\to 0$, the attempt frequency tends toward zero, and $\Delta U_{\mathrm{opt}}\to \infty$. In this limit, the stronger the particle/channel attraction is, the better, as the pore is only rarely occupied by a particle, so that the increase in the translocation probability is the determining factor. In the opposite limiting case, $c_{L}+c_{R}\to \infty$, the attempt frequency tends to infinity, and $\Delta U_{\mathrm{opt}}\to -\infty$, that is, an inverted well. This counter-intuitive result was also obtained [46] within the framework of a discrete stochastic site-binding model of the particle dynamics in the channel.

**Figure 3:**
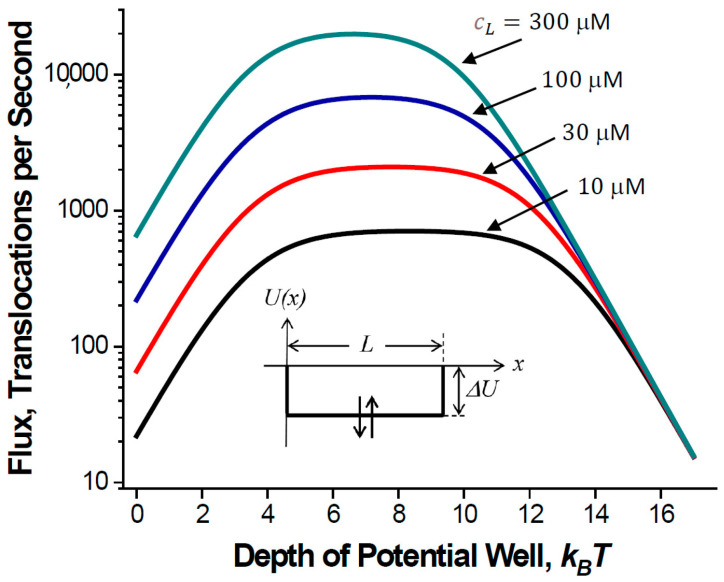
Particle flux as a function of the depth ΔU of a rectangular potential well occupying the entire channel pore (Figure 2C, with l=L). Flux is calculated according to Equation (10) for $c_{R}$ = 0 and other parameters chosen as described in the text.

Channel-facilitated transport through biological membranes typically occurs at large differences in concentrations of translocating molecules on both sides of the membrane. Under such conditions, i.e., when $c_{L}\gg c_{R}$, the non-monotonic behavior of the flux in Equation (10) as a function of the well depth is illustrated in Figure 3. The parameters are chosen to be close to those in real situations, specifically in regard to the equations (i) L = 5 nm, which is approximately the thickness of a lipid bilayer, and (ii) R = 0.2 nm. This radius may seem to be too small, especially if one keeps in mind beta-barrel metabolite channels. However, the model describes molecules as point particles so that the parameter R used in Equation (10) is the difference between the radii of the channel and the molecule. Multiple observations demonstrate that translocating molecules often block the small-ion currents almost completely [4], thus suggesting that the radius of the channel pore is not much larger than that of the molecule: (iii) $D_{b}=2D_{ch}=$ 3 × 10^−10^ m^2^/s, accounting for the fact that a solute in the channel moves slower than in bulk and using the value of a typical bulk diffusion coefficient for metabolite molecules. Figure 3 shows that the optimal well depth is a function of the metabolite concentration; the optimum for 300 μM is about $2k_{B}T$ below that for 10 μM. Importantly, the fluxes predicted by the theory are of the same order of magnitude as those obtained in experiments [4,47].

**Figure 4:**
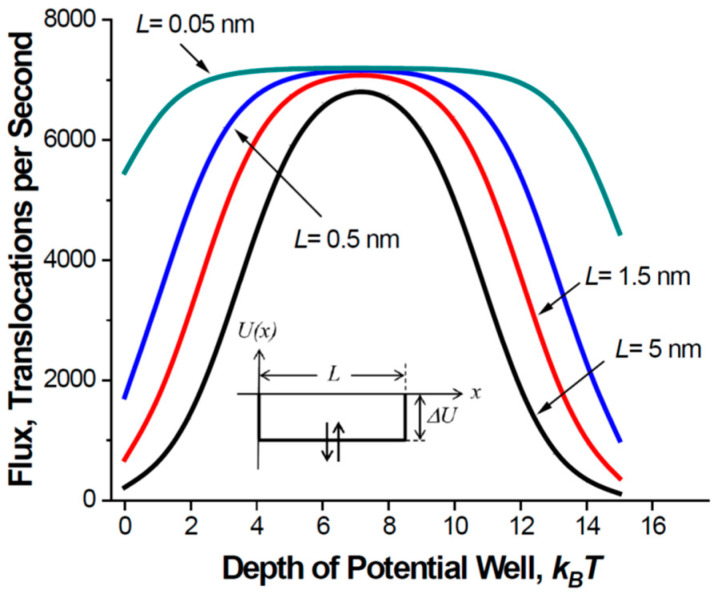
Channel-facilitated flux as a function of the potential well depth for different lengths of the pore *L*. Flux is calculated according to Equation (10) at *c_L_* = 100 μM (6.02 × 10^22^ m^−3^), *c_R_* = 0; other parameters are as those for Figure 3.

The dependence of the optimal well depth on the solute concentration is something to be expected. To be optimal, the channel should be able to release particles quickly enough to be empty by the moment when a next particle arrives. A much less intuitive finding is that the optimal well depth does not depend on the pore length. This follows from Equation (11) and is illustrated by Figure 4, which shows the dependence of the flux on the well depth for channel pores of different lengths. The position of the flux maximum is independent of the pore length. As the length goes to zero, the flux becomes more and more insensitive to the well depth. Only a very strong attraction of the molecules to the channel pore is necessary to decrease the flux. Figure 4 also shows that the pore of a close to zero length is indeed most effective. However, even in the case of a long pore, the optimized interaction increases the flux by orders of magnitude, bringing it close to the flux through the pore of zero length.

**Figure 5:**
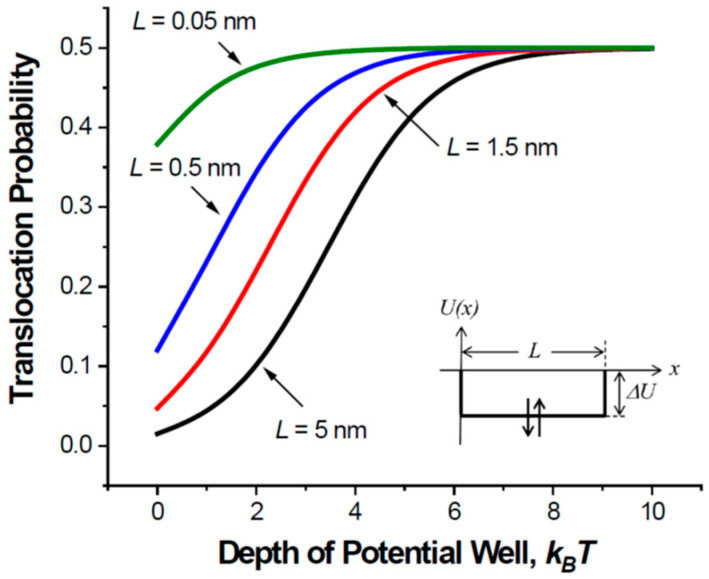
The probability for a particle entering the channel pore to translocate (Equation (7)) as a function of the potential well depth. The parameters are the same as those for Figure 3.

Figure 5 shows the translocation probability given in Equation (7) as a function of the well depth for the same set of parameters as those for Figure 3. A comparison of the two figures reveals the interaction-induced increase in the particle translocation probability that improves channel operation. As the length of the nanopore decreases from 5 nm to 0.5 nm—the latter approximately corresponds to the experimentally determined thickness of a graphene membrane [48]—the flux in Figure 4 and the translocation probability in Figure 5 become less sensitive to the strength of the particle–pore interaction. Analytic results for the unrealistically short pores of length L = 0.05 nm are given only to illustrate the tendency for the cases when the pore length gets smaller than its radius. Short and wide channels provide high translocation probability even in the absence of a potential well. For long and narrow channels, attractive interactions between the particle and the channel pore are crucial, as they increase the initially small translocation probability (e.g., the curve for L = 5 nm at zero well depth) to its absolute maximum of ½.

Our theoretical predictions were confirmed in experiments on single-file diffusion of polystyrene Brownian particles in confining solid-state microchannels where, using attractive optical potentials, the authors were able to create smooth potential wells [49]. They studied potentials extending into the baths and those restricted to the microchannel interior—corresponding to the case analyzed here—and found that, in both cases, the flux of the Brownian particles increased significantly, compared to the microchannel without the attractive potential.

### 2.2. Mean Translocation Time

Consider a symmetric square potential well of length l, l<L, that occupies the central part of the channel pore (Figure 2C):

(12) $$U\left(x\right)=-\Delta UH\left(x-\frac{L-l}{2}\right)H\left(\frac{L+l}{2}-x\right),$$

We introduce λ=l/L as a notation for the fraction of the pore occupied by the well and calculate the mean particle translocation time $\tau_{tr}$. We find that this time turns out to be a non-monotonic function of λ [21]:

(13) $$\tau_{tr}=\tau_{0}\exp\left(\beta \Delta U\right)\frac{Num_{tr}}{2+\mu \left(1-\lambda +\lambda \exp\left(\beta \Delta U\right)\right)},$$

where

(14) $$\begin{array}{l} Num_{tr}=\lambda +\left(1-\lambda \right)\exp\left(-\beta \Delta U\right)+\\ \mu \left[\lambda \left(1-\lambda \right)+\left(1-2\lambda +2\lambda^{2}\right)\exp\left(-\beta \Delta U\right)+\lambda \left(1-\lambda \right)\exp\left(-2\beta \Delta U\right)\right]+\\ \frac{\mu^{2}}{12}\left[3\lambda {\left(1-\lambda \right)}^{2}+2\left(1-\lambda \right)\left(1-2\lambda +4\lambda^{2}\right)\exp\left(-\beta \Delta U\right)+\lambda \left(3-6\lambda +5\lambda^{2}\right)\exp\left(-2\beta \Delta U\right)\right] \end{array}$$

and

(15) $$\tau_{0}=\frac{\pi RL}{4D_{b}},\;\mu =\frac{4LD_{b}}{\pi RD_{ch}}.$$

At λ=0 (l=0), Equation (13) gives the mean particle translocation time through a cylindrical pore of length L and radius R, ${\left.\tau_{tr}\right|}_{\lambda =0}=\tau_{0}\left(1+\mu +\mu^{2}/6\right)/\left(2+\mu \right)$.

**Figure 6:**
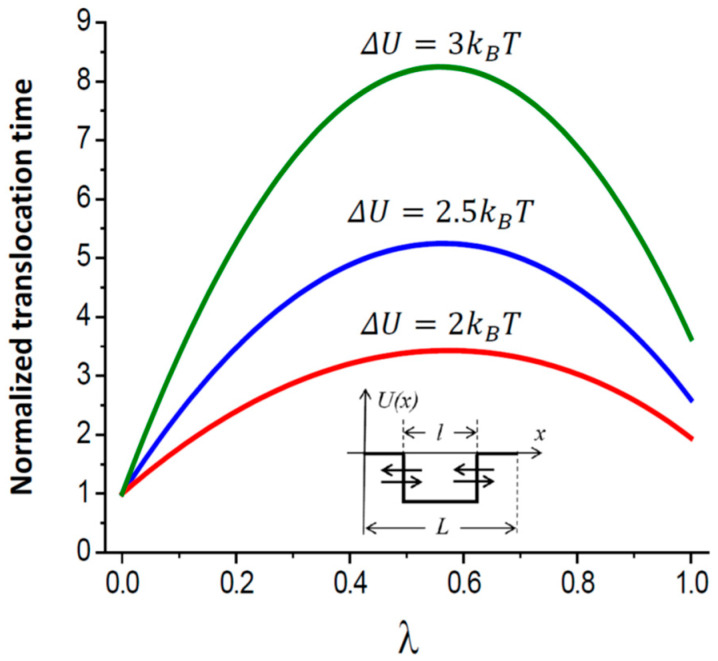
The ratio of the mean translocation time $\tau_{tr}$ in Equation (13) to its value at λ=0 as a function of the fraction of the channel pore occupied by the symmetric square potential well, λ=l/L (Figure 2C), for μ=20, and well depths βΔU = 2, 2.5, and 3 (from bottom to top).

The mean translocation time measured in units of ${\left.\tau_{tr}\right|}_{\lambda =0}$ as a function of λ is displayed in Figure 6 for βΔU=1, 2, and 3 at μ=20. One can check that in the limiting case of a long and narrow channel pore (μ≫1) with a deep potential well (βΔU≫lnμ), the mean translocation time has a maximum at λ=1/2, that is, when the well extends to exactly one-half of the pore, l=L/2. The maximum time is given by the following:

(16) τtrλ=12=τ0μ16exp−βΔU.

Such a non-monotonic behavior of the mean translocation time deserves a qualitative explanation. With this in mind, we note that as the well length tends toward zero, l→0, the well effect on the translocation time vanishes. Correspondingly, an increase in the well length increases this time. It seems reasonable to assume that the process responsible for the increase of this time is the particle recapture by the well. When the well boundaries approach the channel ends, the mean translocation time decreases because of the decrease in the recapture probability. This qualitative consideration suggests the existence of a maximum in the mean translocation time dependence on the well length. Quantitative analytical results in Equations (12)–(15) and Figure 6 confirm this conjecture and show that this maximum is attained at the length l=L/2, that is, when half of the channel length is occupied by the well.

### 2.3. Uphill and Downhill Translocations

Consider now the case when a potential bias is applied to the channel, e.g., U(0)>U(L) in Figure 2B, so that at equal particle concentrations on both sides of the membrane, we have a non-zero net flux of the particles from the left to the right reservoir. We have shown that, rather counter-intuitively, the average direct translocation time in the presence of an arbitrary potential is independent of the direction in which the particle goes [21]. For example, for ions going through a channel pore in the presence of an external voltage, the “uphill” and “downhill” mean direct translocation times are equal notwithstanding that the uphill translocation probability is indeed smaller than the downhill one. Direction invariance of the mean uphill and downhill times has been discussed in Ref. [50] in the context of the escape of overdamped Brownian particles from a deep potential well.

We prove a more general statement that, at an arbitrary bias, not only the mean values, but also the distributions of the uphill and downhill direct translocation times are the same [51]. For the probability densities of the uphill and downhill direct translocation times, assuming that the particle dynamics in the channel are governed by a one-dimensional Langevin equation, we demonstrate the following:

(17) $$\varphi (t|x_{L}\to x_{R})=\varphi (t|x_{R}\to x_{L}),$$

where $x_{L}$ and $x_{R}$ are the coordinates of the left and right channel pore boundaries (Figure 2). The direct translocation time is the time it takes for a particle entering the channel on one side to exit the channel on the opposite side without returning to the reservoir from which it entered initially. This time is a conditional first-passage time; it should not be confused with the unconditional one [52].

The independence of the distribution of the direct translocation time of the passage direction was also shown in a study [53] based on a random walk model of the particle motion in the channel. When the number of sites modeling the channel tends toward infinity and, correspondingly, the interval between successive steps of the random walk tends toward zero in an appropriate way, this model describes the diffusion of the particle in the pore, which is the high-friction limit of the Langevin description of the continuous particle dynamics.

The found identity may be thought of as a direct consequence of microscopic time reversibility. To be more specific, for each trajectory fragment going from left to right, there exists its mirror image going from right to left, which can be obtained by inverting the sign of the particle velocity at each point of the trajectory under consideration.

The identity of the distributions of the direct uphill and downhill translocation times is crucial in constructing a comprehensive theory of counting single-molecule translocations through membrane nanopores. The possibilities of the experimental verification of our results include digital video microscopy of colloidal particles [49] and single-molecule protein folding experiments [54,55]. Such experiments would touch upon Loschmidt’s paradox through direct demonstration that trajectories of a non-equilibrium system are microscopically time reversible, even though the macroscopic system indeed satisfies the Second Law.

### 2.4. Channel Gating as Selectivity Mechanism

Finally, the continuous diffusion model based on the Smoluchowski equation allows one to show that spontaneous gating of the channel pore is able not only to regulate the flux of the particles in the usually accepted way, that is, in proportion to the fraction of time the channel spends in its open conformation, but also to serve as a new mechanism for selectivity [56]. It turns out that if gating is fast enough, it may significantly boost the flux of slower moving particles compared to its conventional predictions. Specifically, we demonstrate that though gating indeed reduces the fluxes of particles of both low and high diffusivity, the interplay of gating and particle intra-channel dynamics favors slower moving particles, as they are able to increase their relative flux by orders of magnitude.

Our motivation for this study comes from the observation that in contrast to the highly ion-selective channels studied in neurophysiology, which are narrow enough to effectively discriminate between partially dehydrated ions [57], metabolite channels are wide because they must accommodate metabolite molecules that typically are much larger than simple mono- or divalent ions. These wide channels may, therefore, act as “shunts” that jeopardize the membrane’s barrier function with respect to small ions. As an example, the most studied metabolite channel, by now the paradigmatic voltage-dependent anion channel (VDAC) [58], has a beta-barrel scaffold of about 1.3 nm inner radius [59,60]. Moreover, being the most abundant integral protein of the outer mitochondrial membrane, it is densely packed in this membrane [61]. Though VDAC is recognized to be the major pathway for ATP and ADP exchange between mitochondria and the cytosol [62], it is also highly permeable for molecules smaller than these metabolites [58]. However, interestingly, in a number of studies, it was demonstrated that under physiological conditions, these channels are predominantly closed [63,64], which allows the membrane to maintain its barrier function. Analyzing the interplay between the channel stochastic gating and particle diffusion dynamics, we proposed that gating may provide a mechanism for metabolite channel selectivity that acts in favor of slower-moving, large solutes.

The stochastic gating of membrane channels is manifested as reversible transitions of channels between their open and closed conformations. To the best of our knowledge, this phenomenon has been known for about a half-century, with the first experimental demonstrations dating back to 1969 to the study by Bean and co-authors [65] who investigated single ion channels produced by the so-called “Excitability Inducing Material” in free-standing lipid bilayers. The gating concept itself is even older and can be traced to the pioneering work by Hodgkin and Huxley [66]. Since then, numerous studies have proved that stochastic gating is the key mechanism by which biological channels respond to the application of transmembrane voltage and to other changes in their environment, which include ligand concentrations, osmotic pressure, temperature, membrane curvature and tension, and membrane lipid composition.

**Figure 7:**
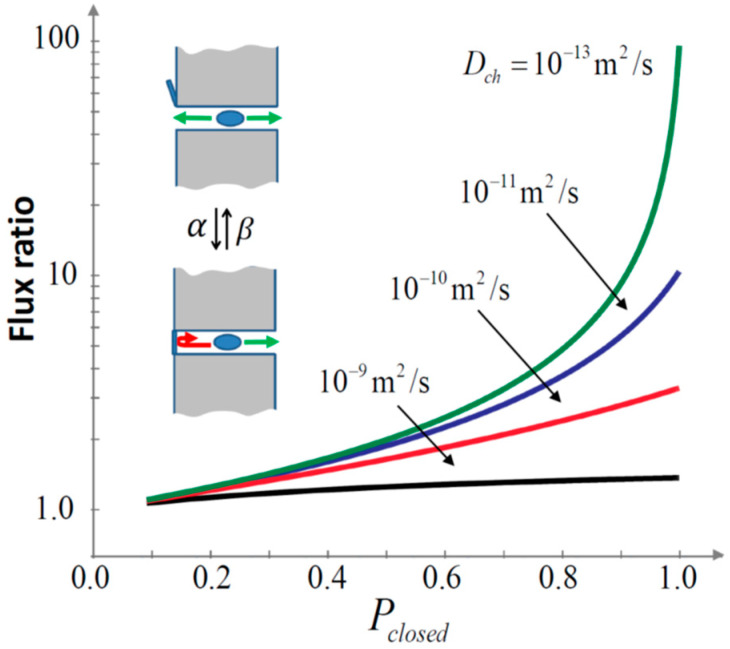
Depending on the molecule intra-channel diffusivity (indicated by the numbers near the curves), the flux through a stochastically gated channel can be significantly different from its conventional estimate. The flux ratio, Equation (19), is the ratio of the flux $J_{g}$, calculated with gating/diffusion interference taken into account its conventional counterpart $J_{g}^{conv}$ given by Equation (18).

In the conventional approach, the flux $J_{g}^{conv}$ through a channel undergoing spontaneous gating is equal to the product of the flux through the open channel $J_{open}$ and the probability of finding the channel in its open state [57,67] $P_{open}=\beta /(\alpha +\beta )$, where α and β are the rate constants of the channel transitions between the open and closed states illustrated in the Figure 7 inset.

Using the continuous diffusion model, we have shown that this simple relation, namely the following:

(18) $$J_{g}^{conv}=J_{open}P_{open},$$

holds true only when the gating dynamics are much slower than particle transport through the channel [56]. When the characteristic times of these processes are close, an interesting and non-trivial effect takes place.

Figure 7 shows the relative increase in the flux of particles due to the channel gating, $J_{g}$, measured in units of the conventional flux estimate $J_{g}^{conv}$, that is, the estimate that obeys Equation (18) and thus does not account for the diffusion interference with gating:

(19) $$\frac{J_{g}}{J_{g}^{conv}}=\frac{2+\kappa L/D_{ch}}{\left(2+\kappa L/D_{ch}\right)\left(1-P_{closed}\right)+P_{closed}F(\lambda )},$$

where

(20) $$F(\lambda )=\frac{2(\kappa L/D_{ch})\lambda +\left[{\left(\kappa L/D_{ch}\right)}^{2}+\lambda^{2}\right]\tanh(\lambda )}{\lambda \left(\kappa L/D_{ch}+\lambda \tanh(\lambda )\right)}$$

with $\lambda =L\sqrt{(\alpha +\beta )/D_{ch}}$, $\kappa =4D_{b}/(\pi R)$, and $P_{closed}=1-P_{open}$. The parameters used for calculating the curves in Figure 7 are as follows: diffusivity in the bulk $D_{b}$ = 2 × 10^−9^ m^2^/s; gating rates are varied with the changing $P_{closed}$ as α = 5 × 10^7^$P_{closed}$ s^−1^ and β = 5 × 10^7^$\left(1-P_{closed}\right)$ s^−1^ to keep the characteristic time of the gating, 1/(α+β), constant for all curves in the figure. Other parameters are L = 4 nm and R = 1.3 nm. It is seen that the molecular flux $J_{g}$, by orders of magnitude, can be significantly higher than the conventional estimate. The effect is strongest when the channel is predominantly closed, i.e., when $P_{closed}$ tends toward 1. The meaning of this finding is that fast gating suppresses transport of faster-moving, small molecules, shown in Equation (18), much stronger than that of slower-moving solutes, but only if the channel spends most of its time in the closed conformation. This represents a newly revealed selectivity mechanism by which the unwanted “shunting” function of large metabolite channels can be minimized. Though presently standing as a hypothesis, we hope that future research on metabolite channel interactions with their cytosolic partners [68] will demonstrate the functional importance of our finding.

Thus, the phenomenon of gating-modified selectivity is based on the interference between the dynamics of channel gating and particle Brownian motion in the channel nanopore. At first glance, it may seem to be similar to the physics involved in Brownian motors action [69,70,71], where randomly fluctuating or periodically changing ratchet structures serve as a mechanism for the selective control over the motion of particles with different diffusivities. Both the Brownian motors and the selectivity mechanism discussed here are based on a modification of particle diffusion by the time-dependent structural changes. However, the mechanism considered here does not require any nonequilibrium, e.g., ATP-dependent, modulation of the structure. While in a Brownian motor, the “detailed balance symmetry, ruling thermal equilibrium dynamics, must be broken by operating the device away from thermal equilibrium” [70], and channel gating is normally caused by equilibrium transitions of the channel-forming protein molecule between different conformations [57,67,72,73,74,75,76].

## 3. Conclusions

Thus, the continuous diffusion model that has been actively developed during last two decades [19,20,21,22,23,24] allows one to analyze channel-facilitated transport in many fine details that are important or, sometimes, even crucial for advancing our understanding of this phenomenon. The model is more realistic than the discrete binding site models of different complexity [10,11,12,13,14,15,16,17,18] because the latter, though providing many illuminating insights into channel functioning, can be viewed as course-grained versions of the continuous diffusion description. At the same time, the continuous diffusion model described here represents, indeed, a minimalistic approach. The crucial step is the drastic simplification in which a wide variety of physical forces acting on a translocating molecule within the nanopore are modeled by a one-dimensional potential of mean force. As stated above, such a simplification is necessary to obtain transparent analytical results, sometimes in the form of simple algebraic expressions.

We started our review with an intuitively appealing result on the probability of particle translocation given by Equation (1), which relates this probability with the geometric parameters of a nanopore. After walking our reader through a number of findings that may seem somewhat unexpected, we would like to finish this review with another intuitively pleasing consequence of our analysis.

Indeed, most of the results obtained within the framework of the continuous diffusion model can be interpreted in terms of macroscopic approaches. Let us demonstrate this for the case of the so-called “diffusion resistance”, $R_{diff}$, which is used to describe transport properties of unstirred layers [77], membranes, and single channels [24] by the following expression for the flux:

(21) $$J=\frac{c_{L}-c_{R}}{R_{diff}}.$$

According to Equation (21), $R_{diff}$ is an analog of electrical resistance, where the concentration difference between left and right compartments, $c_{L}-c_{R}$, plays the role of voltage. Consider the concentration difference-driven flux given by Equation (10) with two simplifying assumptions. First, assume that there are no specific interactions between the particle and the channel pore, ΔU=0, and, second, that the particle concentrations are so small that we can ignore particle–particle interactions, $\pi R^{2}L\left(c_{L}+c_{R}\right)\ll 1$. Then, Equation (10) reduces to the following:

(22) $$J=\;\frac{2D_{b}R\left(c_{L}-c_{R}\right)}{1+\frac{2D_{b}L}{\pi D_{ch}R}}=\;\frac{c_{L}-c_{R}}{\frac{1}{2D_{b}R}+\frac{L}{\pi R^{2}D_{ch}}},$$

which shows that the channel diffusion resistance is as follows:

(23) $$R_{diff}=\frac{1}{2D_{b}R}+\frac{L}{\pi R^{2}D_{ch}}.$$

Equation (23) demonstrates that the channel diffusion resistance is a complete analog of its electrical resistance. Indeed, if one substitutes bulk solution resistivity $\rho_{b}$ for $1/D_{b}$ and intra-channel solution resistivity $\rho_{ch}$ for $1/D_{ch}$, one arrives at the expression for the electrical resistance of a cylindrical channel [57], which is a sum of two access resistances at both entrances of the channel, $\rho_{b}/\left(4R\right)$ each [78], and the resistance of the channel pore proper, $\rho_{b}L/\left(\pi R^{2}\right)$.

Therefore, at least in this simplified case, the solution of the Smoluchowski equation leads to the result that can be obtained from much less sophisticated macroscopic considerations. However, the continuous diffusion model allows one to obtain some straightforward results, which would be hard, if not altogether impossible, to achieve otherwise. As one of the examples, we may point to the phenomenon of gating-induced selectivity discussed in Section 2.4 of the present article. Importantly, the continuous diffusion model goes beyond a macroscopic description to uncover the molecular details of the physics involved in nanopore transport, thus providing a sound theoretical foundation to the growing family of single-molecule methods of modern biophysics.

## Data Availability

The data presented in this study are available upon request from the corresponding author.

## Appendix A

The continuous diffusion model described the propagation of a solute molecule in a cylindrical channel pore of radius R(x) and length L (Figure 2A) in terms of the Green’s function $G(x,t\left|x_{0})\right.$, which was the probability density of finding the molecule at point x at time t, conditional on the idea that it was at $x_{0}$ at t = 0 and had not escaped from the pore during time t. In the case of a channel pore of constant radius, R(x)=R (Figure 1), and in the absence of any specific interactions, the Green’s function satisfied the Smoluchowski equation:

(A1) $$\frac{\partial G(x,t\left|x_{0}\right.)}{\partial t}=D_{ch}\frac{\partial^{2}G(x,t\left|x_{0})\right.}{\partial x^{2}},$$

which was subject to the initial condition $G(x,0\left|x_{0})\right.=\delta (x-x_{0})$ and radiation boundary conditions [19] at the channel pore ends located at x = 0 and x = L,

(A2) $$\begin{array}{l} D_{ch}{\left.\frac{\partial G(x,t\left|x_{0})\right.}{\partial x}\right|}_{x=0}=\kappa G(0,t\left|x_{0})\right.,\\ -D_{ch}{\left.\frac{\partial G(x,t\left|x_{0})\right.}{\partial x}\right|}_{x=L}=\kappa G(L,t\left|x_{0})\right. \end{array}.$$

Here, κ was the trapping rate characterizing the “escape efficiency”, which could be found from the following arguments. Consider a point particle in a system of two compartments connected by a nanopore (Figure 1) at equilibrium. It was equally probable to find the particle at any point and, therefore, the equilibrium density was a constant, $p_{eq}=1/V$, where V was the total volume of the system. The effective one-dimensional density inside the channel pore was also a constant given by $p_{1d,eq}=\pi R^{2}p_{eq}=\pi R^{2}/V$. We determined κ from the condition that fluxes entering and leaving the pore were equal to each other. The flux escaping from the pore into a compartment was $j_{out}=\kappa p_{1d,eq}=\kappa \pi R^{2}/V$. This flux was compensated by the entering flux, $j_{in}=k_{on}/V$, where the rate constant $k_{on}$, given by the following:

(A3) $$k_{on}=4D_{b}R,$$

described the steady-state trapping of particles by an absorbing disk of radius R located on the otherwise reflecting flat wall [43,44]. Since at equilibrium $j_{in}=j_{out}$, we obtained the following:

(A4) $$\kappa =\frac{4D_{b}}{\pi R}.$$

This trapping rate depended on the particle bulk diffusivity $D_{b}$ and was independent of its diffusivity in the channel pore $D_{ch}$. When $D_{b}\to \infty$, the rate diverges, κ→∞, which meant that the pore ends became perfectly adsorbing. In the opposite limiting case, $D_{b}\to 0$, the ends were perfectly reflecting, and the particle never escaped from the channel pore.
